# Randomised β-carotene supplementation and incidence of cancer and cardiovascular disease in women: is the association modified by baseline plasma level?

**DOI:** 10.1038/sj.bjc.6600147

**Published:** 2002-03-04

**Authors:** I-M Lee, N R Cook, J E Manson, J E Buring

**Affiliations:** Division of Preventive Medicine, Department of Medicine, Brigham and Women's Hospital and Harvard Medical School, 900 Commonwealth Avenue East, Boston, Massachusetts, MA 02215, USA; Department of Epidemiology, Harvard School of Public Health, 677 Huntington Avenue, Boston, Massachusetts, MA 02115, USA; Channing Laboratory, Department of Medicine, Brigham and Women's Hospital and Harvard Medical School, 181 Longwood Avenue, Boston, Massachusetts, MA 02115, USA; Department of Ambulatory Care and Prevention, Harvard Medical School, 126 Brookline Avenue, Boston, Massachusetts, MA 02215, USA

**Keywords:** β-carotene, cancer, cardiovascular disease, epidemiology

## Abstract

In a nested case-control study of 513 women with cancer; 130 with cardiovascular disease and equal numbers of controls, we found no effect of randomised beta-carotene on risk of cancer or cardiovascular disease within any quartile of baseline plasma beta-carotene, nor was there a trend across quartiles (*P* for trend 0.15 and 0.62, respectively).

*British Journal of Cancer* (2002) **86**, 698–701. DOI: 10.1038/sj/bjc/6600147 www.bjcancer.com

© 2002 Cancer Research UK

## 

Observational epidemiologic studies have consistently indicated that individuals who consume large amounts of fruits and vegetables rich in β-carotene experience lower cancer rates (e.g., Van Poppel, 1996; Toniolo *et al*, 2001). However, randomised trials testing β-carotene supplementation, alone or in combination with other supplements, have not supported this (Greenberg *et al*, 1990; Blot *et al*, 1993; Li *et al*, 1993; The Alpha-Tocopherol, Beta Carotene Cancer Prevention Study Group, 1994; Hennekens *et al*, 1996; Omenn *et al*, 1996b; Lee *et al*, 1999). Of seven trials, one observed a significant benefit on cancer mortality (Blot *et al*, 1993), four reported no significant benefit or harm on the incidence of cancer and cardiovascular disease (Greenberg *et al*, 1990; Li *et al*, 1993; Hennekens *et al*, 1996; Lee *et al*, 1999), while the remaining two trials found an unexpected, but significant increase in lung cancer incidence (Omenn *et al*, 1996b; The Alpha-Tocopherol, Beta Carotene Cancer Prevention Study Group, 1994). The only trial reporting a benefit of β-carotene supplementation tested a combination of beta-carotene, vitamin E, and selenium among poorly nourished adults in China (Blot *et al*, 1993). This has raised the hypothesis that any benefit of β-carotene supplementation may be limited to those with low levels of plasma β-carotene. In this paper, we conduct a *post-hoc* analysis to test the hypothesis using data from the Women's Health Study (WHS), where we previously observed no overall benefit or harm from β-carotene on cancer and cardiovascular disease (Lee *et al*, 1999).

## MATERIALS AND METHODS

The WHS was originally designed as a randomised, double-blind, placebo-controlled trial testing the balance of benefits and risks of aspirin (Bayer AG, Leverkusen, Germany), vitamin E (Natural Source Vitamin E Association, Washington, DC, USA), and β-carotene (Lurotin, 50 mg every other day; BASF Corporation, Wyandotte, MI, USA) in the primary prevention of cancer and cardiovascular disease among 39 876 female health professionals aged ⩾45 years (Buring and Hennekens, 1992a,b; Rexrode *et al*, 2000). Written informed consent was obtained from the women, and the trial was approved by the institutional review board of Brigham and Women's Hospital (Boston, MA, USA). The β-carotene component of the trial was terminated early on January 18, 1996 after a median treatment duration of 2.1 years, based on the null findings in the Physicians' Health Study (Hennekens *et al*, 1996) and also because two other trials had suggested increased risk of lung cancer (The Alpha-Tocopherol, Beta Carotene Cancer Prevention Study Group, 1994; Omenn *et al*, 1996b). (The aspirin and vitamin E components are currently ongoing.)

Prior to randomisation, women were asked to provide baseline blood samples; 28 133 (71%) did so. Plasma samples were stored at −170°C. The occurrence of cancer and cardiovascular disease was ascertained using questionnaires sent twice in the first year and yearly thereafter. Deaths were reported by family members or postal authorities. We obtained medical records to confirm the occurrence of cancer and cardiovascular events. After a median follow-up of 4.1 years, 747 confirmed cases of cancer and 218 of cardiovascular disease (non-fatal myocardial infarction, non-fatal stroke, or cardiovascular death) had occurred. Of these women with cancer and cardiovascular disease, 513 and 130, respectively, had provided baseline blood samples; they represent the cases for the present nested case–control study. For each case, we selected a control subject who had provided a baseline blood sample and had no cancer or cardiovascular disease up to the time of diagnosis of the case. Controls also were matched on age (within 1 year), smoking habit, and time since randomisation (within 6 months).

We measured plasma β-carotene by high performance liquid chromatography using Shimadzu system (De Leenheer *et al*, 1979). This assay was standardised using calibrators from the National Institute of Standards and Technology. The intra- and inter-assay coefficients of variation were <4.2% and <6.1%, respectively. Plasma samples from each case and matching control were assayed in random order within the same batch to minimise inter-assay variability, and laboratory personnel were blinded to the case or control status of samples.

We compared the baseline characteristics of cases and controls using stratified analysis of matched sets. Proportions were compared with the stratified Cochran–Mantel Haenszel test; means, with models allowing for the correlation of values within matched sets, using PROC MIXED of SAS (SAS Institute Inc, 1997). We then categorised women into quartiles of plasma β-carotene at baseline. Conditional logistic regression analysis was performed using Cox proportional hazards models stratified by matched set (Breslow and Day, 1980). Models were additionally adjusted for randomised aspirin and vitamin E. To evaluate the joint association of baseline β-carotene and randomised β-carotene, models were fit with interaction terms between quartile of baseline plasma β-carotene level and randomised β-carotene assignment.

## RESULTS

The baseline characteristics of cancer cases and their matched controls are shown in Table 1

**Table 1:**
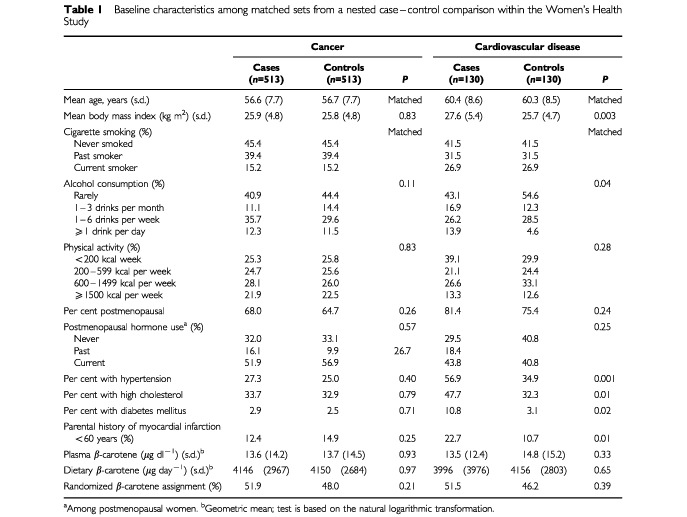
Baseline characteristics among matched sets from a nested case–control comparison within the Women's Health Study

. The mean age was 56.7 years. Just over 15% of women were smoking cigarettes at baseline. Cancer cases and controls did not differ significantly in body mass index, alcohol intake, physical activity, menopausal status, or use of postmenopausal hormones. At baseline, they had very similar plasma levels of β-carotene (13.6 and 13.7 μg dl^−1^, respectively) and dietary intake of β-carotene (4146 and 4150 μg day^−1^). Similar proportions were randomised to active β-carotene (51.9 and 48.0%).

Women with cardiovascular disease and their matched controls were older at baseline, with a mean age over 60 years, and included more current smokers (26.9%). Women with cardiovascular disease were heavier than controls; drank more alcohol; and were more likely to have a personal history of hypertension, high cholesterol, and diabetes mellitus, and a parental history of early myocardial infarction. However, they did not differ in physical activity, postmenopausal status, or use of postmenopausal hormones. They also did not differ significantly in baseline levels of plasma β-carotene (13.5 and 14.8 g dl^−1^, respectively), dietary intake of β-carotene (3996 and 4156 μg day^−1^), and proportion randomised to active β-carotene (51.5 and 46.2%).

Table 2

**Table 2:**
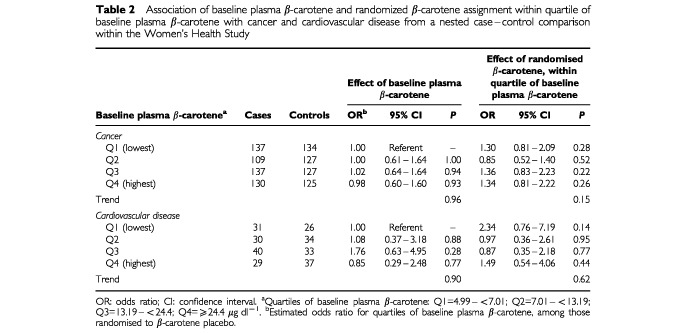
Association of baseline plasma β-carotene and randomized β-carotene assignment within quartile of baseline plasma β-carotene with cancer and cardiovascular disease from a nested case–control comparison within the Women's Health Study

shows the results from conditional logistic regression analysis of the associations of both plasma β-carotene level at baseline and randomised β-carotene assignment with cancer and cardiovascular disease. For cancer, there was no evidence that higher plasma levels were related to decreased risk (odds ratio (OR) comparing highest *vs* lowest quartile among women assigned placebo β-carotene=0.98; 95% confidence interval (CI)=0.60–1.60), and the trend in risk across quartiles was nonsignificant (*P*, trend=0.96). We next examined the effect of randomised β-carotene supplementation, within quartiles of baseline plasma β-carotene levels. There was no effect of randomised β-carotene in any quartile, nor was there a trend across quartiles (*P*, trend=0.15). Additional adjustment for body mass index, alcohol intake, physical activity, menopausal status, and use of postmenopausal hormones did not change these findings (data not shown).

For cardiovascular disease, a higher plasma level of β-carotene at baseline also was not predictive of lower risk (corresponding OR=0.85; 95% CI=0.29–2.48; *P*, trend=0.90). As with cancer, there was no effect of randomised β-carotene in any quartile (*P*, trend=0.62). Additional adjustment for the potential confounders above, plus personal history of hypertension, high cholesterol, and diabetes mellitus, and a parental history of early myocardial infarction did not change our conclusions (data not shown).

## DISCUSSION

Previous analyses of all 39 876 women in the WHS revealed no overall benefit or harm of randomised β-carotene supplementation on cancer or cardiovascular disease, after a median treatment duration of 2.1 years and a median follow-up of 4.1 years (Lee *et al*, 1999). In the present case–control study nested within the WHS, the data further show that no effect is observed, regardless of baseline plasma β-carotene level.

In concert with these findings, the Alpha-Tocopherol, Beta-Carotene Cancer Prevention (ATBC) Study reported no effect of baseline serum β-carotene on the association between randomised β-carotene supplementation and lung cancer risk (Albanes *et al*, 1996). Likewise, the Beta-Carotene and Retinol Efficacy Trial (CARET) observed the same relative risk for lung cancer among those assigned a combination of β-carotene and retinyl palmitate supplements *vs* placebo, regardless of whether baseline serum β-carotene was below or above the median (Omenn *et al*, 1996a). In contrast, the Physicians' Health Study (PHS) reported that the effect of randomised β-carotene on cancer risk was marginally different (*P*, trend=0.09) across quartiles of baseline plasma β-carotene levels, with the largest benefit seen in the lowest quartile (Cook *et al*, 1999). This trend was primarily due to a benefit for prostate cancer risk, which may explain the different finding from the WHS.

The present findings do not necessarily refute the hypothesis that β-carotene supplementation may be beneficial to those with low levels of plasma β-carotene. In the Chinese study where decreased cancer mortality was observed among individuals randomly assigned a combination of β-carotene, vitamin E and selenium, the mean baseline blood level of β-carotene was 5.9–6.8 μg dl^−1^ (Blot *et al*, 1993). This was higher in the WHS (13.7–14.8 μg dl^−1^), as well as in the three other trials conducted among well-nourished populations (ATBC study: median, 17 μg dl^−1^; 20th–80th percentile, 10–29 μg dl^−1^ (The Alpha-Tocopherol, Beta Carotene Cancer Prevention Study Group, 1994); CARET: median, 15.2 μg dl^−1^ (Omenn *et al*, 1996a); PHS: mean among men free of cancer, 22.5 μg dl^−1^; 25th–75th percentile, 15.3–34.4 μg dl^−1^ (Cook *et al*, 1999)). Therefore, the difference in findings among these trials may reflect the different nutritional status of the subjects.

Strengths of the present study include good compliance with randomised treatment assignment (Lee *et al*, 1999) and careful documentation of endpoints. A major limitation, however, is that randomised treatment with β-carotene lasted only 2.1 years before early termination, so we could not evaluate long-term treatment. Follow-up duration was also limited; the relatively few cases of cardiovascular disease led to findings with wide confidence intervals. Another concern is that only 71% of WHS women provided blood samples at baseline and analyses were limited to women with a baseline blood sample. However, adjustment for multiple risk factors for cancer and cardiovascular disease, which might be related to baseline blood contribution, did not change the results.

In conclusion, these data do not suggest that β-carotene supplementation is beneficial to individuals with the lowest plasma levels of β-carotene as found within this study. However, because subjects were adequately nourished, we could not examine this question among those with levels of β-carotene that might be seen in a malnourished population. Further, treatment duration was short, precluding the evaluation of long-term supplementation.
